# Active screening and molecular epidemiological characteristics of fecal colonization by carbapenem-resistant *Enterobacterales* in intensive care unit wards of a tertiary hospital in Shanghai, China

**DOI:** 10.3389/fpubh.2025.1668856

**Published:** 2025-11-07

**Authors:** Hui Zhang, Cong Zhou, Maosuo Xu, Chunmei Shen, Fang Shen, Yong Lin

**Affiliations:** 1 Department of Clinical Laboratory, Shanghai Fifth People’s Hospital, Fudan University, Shanghai, China; 2 Center of Community-Based Health Research, Fudan University, Shanghai, China

**Keywords:** active screening, fecal colonization, carbapenem-resistant *Enterobacterales*, molecular epidemiological characteristics, homology

## Abstract

**Background:**

Active screening for fecal colonization by carbapenem-resistant *Enterobacterales* (CRE-FC) and interventions in intensive care unit (ICU) wards have become important measures to prevent carbapenem-resistant *Enterobacterales* (CRE) infection. However, limited data are available on the molecular epidemiology and homology analysis of CRE-FC. This study tried to investigate the molecular epidemiology characteristics and homology of CRE-FC in ICU wards to provide evidence for CRE transmission.

**Methods:**

From March 1, 2022 to February 28, 2023, fecal swabs from 435 ICU patients were analyzed using resistant bacteria chromogenic plates. Duplicate strains from the same patient were excluded. Infection prevention and control (IPC) measures were implemented for patients with positive CRE screening results. Bacterial identification, antimicrobial susceptibility testing, multilocus sequence typing (MLST), capsule serotypes, whole-genome sequencing (WGS), and core-genome MLST (cgMLST) were conducted to analyze the molecular epidemiological features and homology of these strains.

**Results:**

The prevalence of CRE-FC in ICU wards was 12.6% (55/435). The predominant CRE-FC was *Klebsiella pneumoniae* (83.6%, 46/55) and *Escherichia coli* (9.1%, 5/55). Active screening and IPC interventions in 2022 reduced the CRE infection rate decreased from 11.4 to 7.1%. MLST analysis of 46 carbapenem-resistant *K. pneumoniae* (CRKP-FC) strains showed that ST11 was the dominant sequence type (71.7%, 33/46), followed by ST15 (26.1%, 12/46) and ST290 (2.2%, 1/46). All ST11 and ST15 strains carried *bla*_KPC-2_, 10 of the ST15 strains additionally carried both *bla*_KPC-2_ and *bla*_OXA-1_. Phylogenetic analysis revealed two major clades: ST11 and ST15.

**Conclusion:**

CRKP-FC was predominant in CRE-FC. Furthermore, phylogenetic analysis suggested that CRKP-FC had clonal spread in ICU wards, with ST11-KL64 as the main clone. Active CRE-FC screening and IPC interventions can effectively reduced CRE infection rates.

## 1 Introduction

Carbapenem-resistant *Enterobacterales* (CRE) infection has emerged as a major global public health threat and has caused serious societal and economic burdens (1, 2). Notably, carbapenem-resistant *K. pneumoniae* (CRKP) account for 60–90% of clinical CRE infections in the United States, Europe, and China (3–5). According to the 2023 China Antimicrobial Surveillance Network (CHINET), the resistance rates of *K. pneumoniae* to meropenem have increased from 2.9% in 2005 to 30.0% in 2023 (6). Moreover, CRKP is dominant in the intestines colonized with CRE (7, 8). CRE colonization is an important risk factor for the development of CRE infection in hospitals and communities (9–12).

*Enterobacterales* develop resistance to carbapenems through two main mechanisms. Non-carbapenemase-producing carbapenem-resistant *Enterobacterales* (via one or a combination of alterations in membrane permeability, alterations in drug efflux pumps, and alterations in antimicrobial target site binding) and carbapenemase-producing carbapenem-resistant *Enterobacterales* (e.g., KPC, NDM, VIM and OXA) (13, 14). Carbapenemase-producing *Enterobacterales* (CPEs) (15), which have relatively high levels of antimicrobial resistance, clonal spread and horizontal gene transfer, are of greatest concern (16).

Studies have revealed that the CRE colonization rate of patients in ICU wards is higher than that of patients in other wards (7, 17). Moreover, the CRE infection rate of patients in ICU is also higher than that of patients in other wards (18, 19). Other studies have shown that active screening of CRE-FC and IPC interventions can reduce the CRE infection rate in ICU wards (20, 21).

Active screening of CRE-FC in ICU wards has become an important prevention measure for CRE infection (22). However, limited data are available on the molecular epidemiological characteristics and homology analysis of CRE-FC in ICU wards. To gain deeper insights into the molecular epidemiological characteristics and homology of CRE-FC in ICU wards, we employed advanced analytical methods including whole-genome sequencing (WGS) and core-genome multilocus sequence typing (cgMLST), thereby filling a gap in previous research in this field. Exploring the molecular epidemiological characteristics and homology analysis of CRE-FC in ICU wards can provide evidence for CRE transmission, which is highly valuable for improving the application of infection control measures and reducing mortality. This prospective observational study aimed to investigate the molecular epidemiological characteristics and homology of CRE-FC in ICU wards of a university hospital in China.

## 2 Materials and methods

### 2.1 Study design

Fecal swabs were collected from 435 patients in the ICU wards of a tertiary hospital in Shanghai, China, from March 1, 2022, to February 28, 2023, and the above-mentioned samples were inoculated in a Resistant Bacteria Chromogenic Plate (Antu Bio, China). At admission to the ICU wards, fecal swabs were performed by a trained nurse, and the swabs were immediately sent to the clinical laboratory. Meanwhile, to monitor changes in CRE colonization, fecal swabs were repeated twice a week during hospitalization. Each sample was accompanied by a case report form containing the clinical characteristics of the patients, including name, sex, age, admission time, temperature, diagnosis, antibiotic used and laboratory test results. Therefore, active screening of CRE-FC was performed. The meropenem-resistant colonies were subsequently selected, and bacterial identification was performed via MALDI-TOF mass spectrometry (Bruker Daltonics, Billerica, MA, Germany), and antimicrobial susceptibility testing was carried out using a VITEK-2 compact automated microbiology analyser (Biomerieux, Marcy L‘Etoile, France). We removed duplicate strains from the same patient and retained only the first isolated CRE-FC. By quality control, strains were excluded due to low-quality assembly as defined by NCBI (e.g., excessive frameshifted proteins and fragmented assembly), <95% completeness, >5% contamination, or genomes belonging to species other than CRE. A total of 55 isolates of fecal colonization with CRE were identified. Two strains of CRKP, WYZHKP101 and WYZHKP102, were isolated from the ICU environment for comparison with CRKP-FC. The CRE strains were used to test for the presence of the carbapenemase genes *bla*_KPC_, *bla*_NDM_, *bla*_VIM_, *bla*_IMP_, and *bla*_OXA-48_ via PCR with specific primers (*bla*_KPC_: forward: ATGTCACTGTATCGCCGTCT, reverse: TTTTCAGAGCCTTACTGCCC; *bla*_NDM_: forward: GGTTTGGCGATCTGGTTTTC, reverse: CGGAATGGCTCATCACGATC; *bla*_VIM_: forward: GATGGTGTTTGGTCGCATA, reverse: CGAATGCGCAGCACCAG; *bla*_IMP_: forward: GGAATAGAGTGGCTTAAYTCTC, reverse: GGTTTAAYAAAACAACCACC; *bla*_OXA-48_: forward: GCGTGGTTAAGGATGAACAC, reverse: CATCAAGTTCAACCCAACCG).

### 2.2 Antimicrobial susceptibility testing

Antimicrobial susceptibility testing was performed via the broth microdilution method of the Clinical and Laboratory Standards Institute (CLSI). The results were interpreted according to 2022 CLSI breakpoints (23) for all antimicrobial agents except tigecycline, which was interpreted via the interpretation standards of the US Food and Drug Administration (FDA). *E. coli* ATCC 25922 and *K. pneumoniae* ATCC 700603 were used as controls for antimicrobial susceptibility testing.

### 2.3 Whole-genome sequencing and bioinformatics analysis

As the predominant CRE-FC was *K. pneumoniae* (83.6%, 46/55), it is necessary to further analyze the phenotype and genome of 46 CRKP-FC strains. Two CRKP strains, WYZHKP101 and WYZHKP102, which were isolated from the environment, were also further analyzed for phenotype and genome homology. Finally, 48 CRKP isolates were selected and subjected to whole-genome sequencing (WGS). The genomic DNA of the *K. pneumoniae* strains was extracted via a commercial DNA extraction kit (Qiagen, Germany) following the manufacturer’s instructions. Fragmented genomic DNA was sequenced via the 150-bp paired-end Illumina HiSeq platform (Illumina, San Diego, CA, United States). *De novo* assembly was conducted via Unicycler version 0.4.8 (24). Then, the genome sequence was acquired after rectification via pilon software (25). Gene annotation was performed via Prokka version 1.12 (26).

Antimicrobial resistance genes were identified via ResFinder 4.1^1^. Multilocus sequence typing (MLST), capsule serotypes and virulence genes were identified according to BIGSdb-Pasteur^2^. Core genome multilocus sequence typing (cgMLST) analysis of strains was performed by comparing the 2,358 conserved genome-wide genes of *K. pneumoniae* using the software Ridom SeqSphere+ version 9.0.8 (Ridom GmbH, Muenster, Germany), with which a phylogenetic tree was constructed. The distance of ≤15 alleles is defined as a cluster. (27, 28).

### 2.4 Phylogenetic analysis

We used single-nucleotide polymorphisms (SNPs) to investigate the transmission of *K. pneumoniae* based on genome sequences mining. The phylogenetic analysis included 48 *K. pneumoniae* genomes from this study and 185 *K. pneumoniae* genomes obtained from National Center for Biotechnology Information (NCBI). These 233 genome assemblies were subjected to removing recombination regions using Gubbins^3^ and thereafter to calling core SNPs with Snippy^4^ using strain WYZHKP18 (accession no. GCA_040117795.1) as the reference. We subsequently visualized and annotated a phylogenetic tree using Interactive Tree Of Life (iTOL)^5^.

### 2.5 IPC interventions and effect evaluation

In accordance with the World Health Organization (WHO) guidelines (29), IPC interventions were carried out for patients with positive CRE screening results. These interventions included contact isolation, the use of disposable or dedicated patient care equipment, and strict enforcement of hand hygiene by medical staff and wards disinfection. The hand hygiene compliance rate is calculated as the ratio of the number of times hand hygiene is performed correctly to the total number of opportunities for hand hygiene, multiplied by 100% to get a percentage value. It reflects the adherence of medical staff to proper hand hygiene practices, which is essential for preventing the spread of infections. Additionally, the wards were subjected to disinfection to maintain environmental hygiene. The environmental hygiene compliance rate is determined by dividing the number of environmental hygiene parameters that meet the set standards by the total number of parameters tested and expressing it as a percentage. This helps in assessing the cleanliness and safety of the ward environment.

The CRE infection rate refers to the proportion of individuals infected with CRE (carbapenem-resistant Enterobacteriaceae) among the specific population or in a specific environment, usually expressed as a percentage. The calculation formula is: CRE infection rate = (the number of individuals infected with CRE/the total number of individuals examined) × 100%. The CRE infection rate can mirror the infection situation of CRE within particular populations or settings, and contribute to assessing the evaluation metrics for the effectiveness of CRE positive screening and IPC interventions. The CRE infection rates in ICU wards were compared between the periods without active CRE screening and IPC interventions (from March 1, 2022, to February 28, 2023) and the periods with active CRE screening and IPC interventions (from March 1, 2022, to February 28, 2023).

### 2.6 CRE definition

The CRE was defined as carbapenem-resistant *Enterobacterales* including CRKP, carbapenem-resistant *E. coli* (CRECO) and other bacteria were resistant to at least one of the carbapenems, including imipenem, meropenem, and ertapenem.

### 2.7 Statistical analysis

The data are presented as the means ± standard deviations (SDs). All the statistical analyses were performed via SPSS version 23.0 (IBM Co., Armonk, NY, United States). Categorical variables were assessed by the chi-square test or Fisher’s exact test. All the statistical tests were 2 tailed, and *p* < 0.05 was considered statistically significant.

## 3 Results

### 3.1 Fecal carriage of carbapenem-resistant *Enterobacterales*

**Figure 1:**
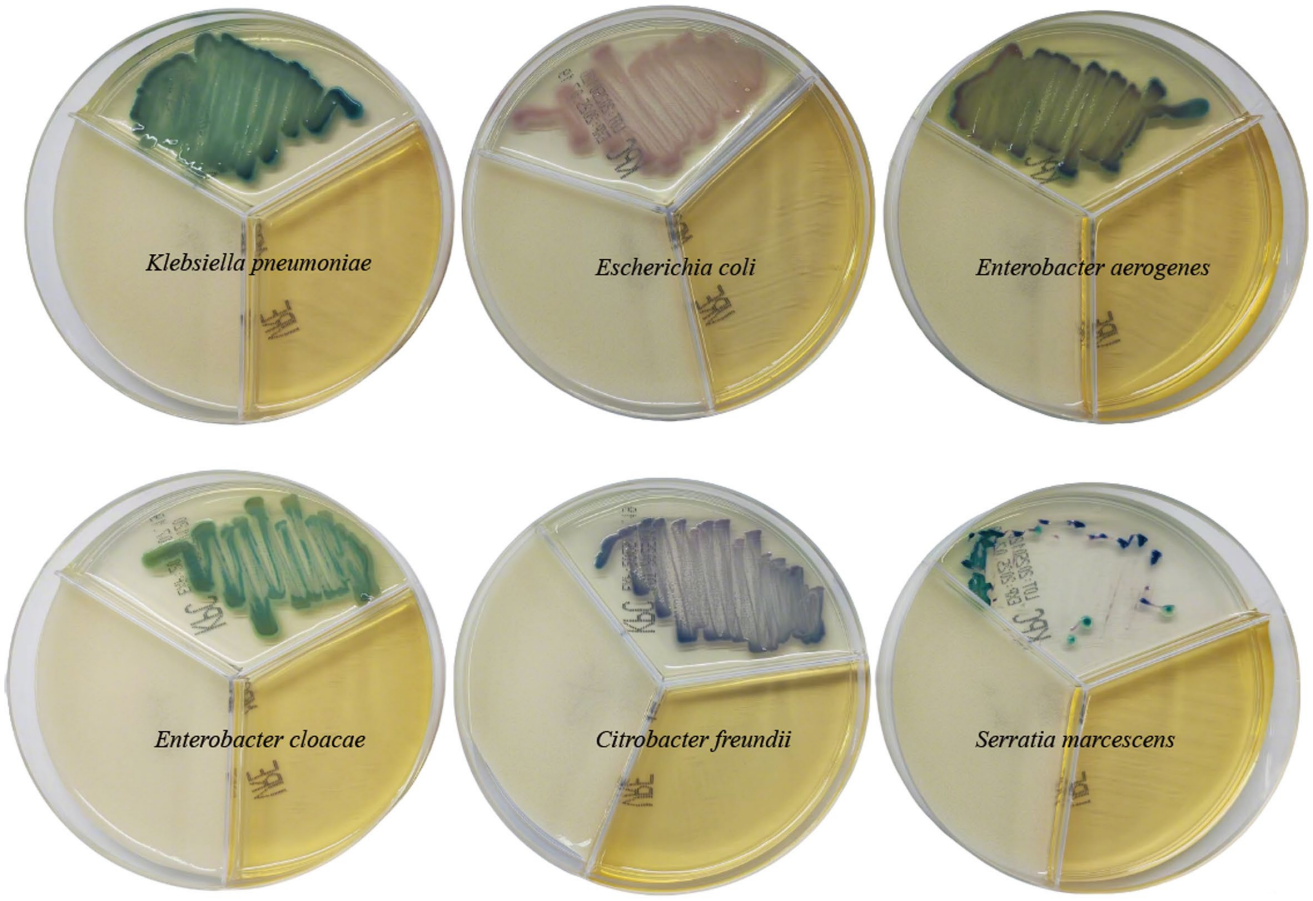
The coloration of different carbapenem-resistant enterobacterales on resistant bacteria chromogenic plate.

During the study period, we actively screened fecal swabs from 435 patients in ICU wards (Figure 1). The prevalence of CRE-FC in ICU wards was 12.6% (55/435). The predominant CRE-FC was *K. pneumoniae* (83.6%, 46/55), followed by *E. coli* (9.1%, 5/55), *Klebsiella aerogenes* (3.7%, 2/55), *Enterobacter cloacae* (1.8%, 1/55), and *Citrobacter freundii* (1.8%, 1/55). We removed duplicate strains from the same patient and retained only the first isolated CRE-FC. The 55 CRE-FC strains were isolated from 55 individual patients (35 males and 20 females), whose mean age was 70.6 ± 13.5 years. Two strains of CRKP, WYZHKP101 and WYZHKP102, were isolated from the ICU environment for comparison with CRKP-FC. As the predominant CRE-FC was *K. pneumoniae* (83.6%, 46/55), further analysis of the phenotype and genome of 46 CRKP-FC strains, WYZHKP101 and WYZHKP102, is necessary.

### 3.2 Molecular characterization of carbapenemase genes

The distribution of carbapenemase genes of 55 CRE-FC strains is shown in Table 1. As shown in Table 1, the most prevalent carbapenemase gene was *bla*_KPC_ (89.1%, 49/55), which was detected in 45 *K. pneumoniae* isolates, 1 *E. coli*, 2 *Klebsiella aerogenes* and 1 *C. freundii*, followed by *bla*_NDM_ (10.9%, 6/55), which was detected in 4 *E. coli* isolates, 1 *K. pneumoniae* isolate and 1 *E. cloacae isolate*; *bla*_IMP_, *bla*_VIM_, and *bla*_OXA-48_ were not detected (Table 1).

**Table 1:** Carbapenemase genes in 55 CRE-FC strains.

Bacteria	Carbapenemase genes (isolate number)				
	*bla*_KPC_	*bla*_NDM_	*bla*_VIM_	*bla*_IMP_	*bla*_OXA-48_
*K. pneumoniae*	45	1	–	–	–
*E. coli*	1	4	–	–	–
*K. aerogenes*	2	–	–	–	–
*E. cloacae*	–	1	–	–	–
*C. freundii*	1	–	–	–	–

### 3.3 Active screening of CRE-FC and IPC interventions reduces the CRE infection rate in ICU wards

The data presented in Table 2 reveals that in comparison to 2021, the timely isolation rate of CRE-positive patients, the hand hygiene compliance rate, and the qualified rate of environmental disinfection all showed improvements. When it comes to resistance rates, variations were observed among different types of CRE. Specifically, the carbapenem resistance rate of *K. pneumoniae* in 2022 (39.3%) was significantly lower than that in 2021 (55.9%), with a statistically significant difference. In contrast, the carbapenem resistance rate of *E. coli* remained relatively stable, being 2.9% in 2022 and 2.6% in 2021, and the difference was not statistically significant. Additionally, the CRE infection rate decreased from 11.4 to 7.1%. Overall, the data indicates that active screening of CRE-FC and the implementation of IPC interventions can effectively reduce the CRE infection rate in ICU wards.

**Table 2:** Actively screen CRE-FC results and IPC intervention implementation data in ICU wards from 2021 to 2022.

Year	Timely isolation rate of CRE positive patients (%)	The hand hygiene compliance rate (%)	Qualified rate of environmental disinfection (%)	CRE infection rate (%)	Resistance rates (%)			
					CRE	CRKP	CRECO	Other
2021	94.7 (177/187)	90.0 (325/361)	95.4 (103/108)	11.4 (76/667)	33.7 (63/187)	55.9 (57/102)	2.6 (1/38)	10.6 (5/47)
2022	99.0 (302/305)	94.7 (571/603)	99.2 (262/264)	7.1 (31/435)	22.8 (57/250)	39.3 (48/122)	2.9 (2/69)	11.8 (7/59)
*p*-value	0.003	0.006	0.013	0.041	0.012	0.014	0.936	0.844

CRE, carbapenem-resistant *Enterobacterales*; CRKP, carbapenem-resistant *K. pneumoniae*; CRECO, carbapenem-resistant *E. coli*.

### 3.4 Antimicrobial resistance profile of 46 carbapenem-resistant *K. pneumoniae* strains

To clarify the antibiotic resistance profile of 46 CRKP strains colonized via feces, we tested their susceptibility to 18 antibiotics (Table 3). All the CRKP strains presented multiple drug resistance phenotypes and were resistant to three or more antibiotic classes. The 46 CRKP-FC strains were resistant to ampicillin, ampicillin/sulbactam, cefoperazone/sulbactam, piperacillin/tazobactam, cefazolin, cefuroxime, ceftazidime, ceftriaxone, cefepime, cefoxitin, imipenem, and meropenem. The antimicrobial resistance rate of ceftazidime/avibactam was the lowest (6.5%), followed by tigecycline (8.7%), trimethoprim-sulfamethoxazole (73.9%), amikacin (76.1%), gentamicin (93.5%) and levofloxacin (97.8%).

**Table 3:** Antibiotic susceptibilities of 46 carbapenem-resistant *K. pneumoniae* strains.

Antibiotics	Sensitivity (%)	Intermediate (%)	Resistance (%)
Ampicillin	0	0	100
Ampicillin/sulbactam	0	0	100
Cefoperazone/sulbactam	0	0	100
Piperacillin/tazobactam	0	0	100
Cefazolin	0	0	100
Cefuroxime	0	0	100
Ceftazidime	0	0	100
Ceftriaxone	0	0	100
Cefepime	0	0	100
Cefoxitin	0	0	100
Imipenem	0	0	100
Meropenem	0	0	100
Amikacin	21.7	2.2	76.1
Gentamicin	6.5	0	93.5
Levofloxacin	2.2	0	97.8
Trimethoprim-sulfamethoxazole	26.1	0	73.9
Ceftazidime/Avibactam	93.5	0	6.5
Tigecycline	76.1	15.2	8.7

### 3.5 Distribution of antibiotic resistance genes, virulence genes, MLST and capsular serotypes

**Figure 2:**
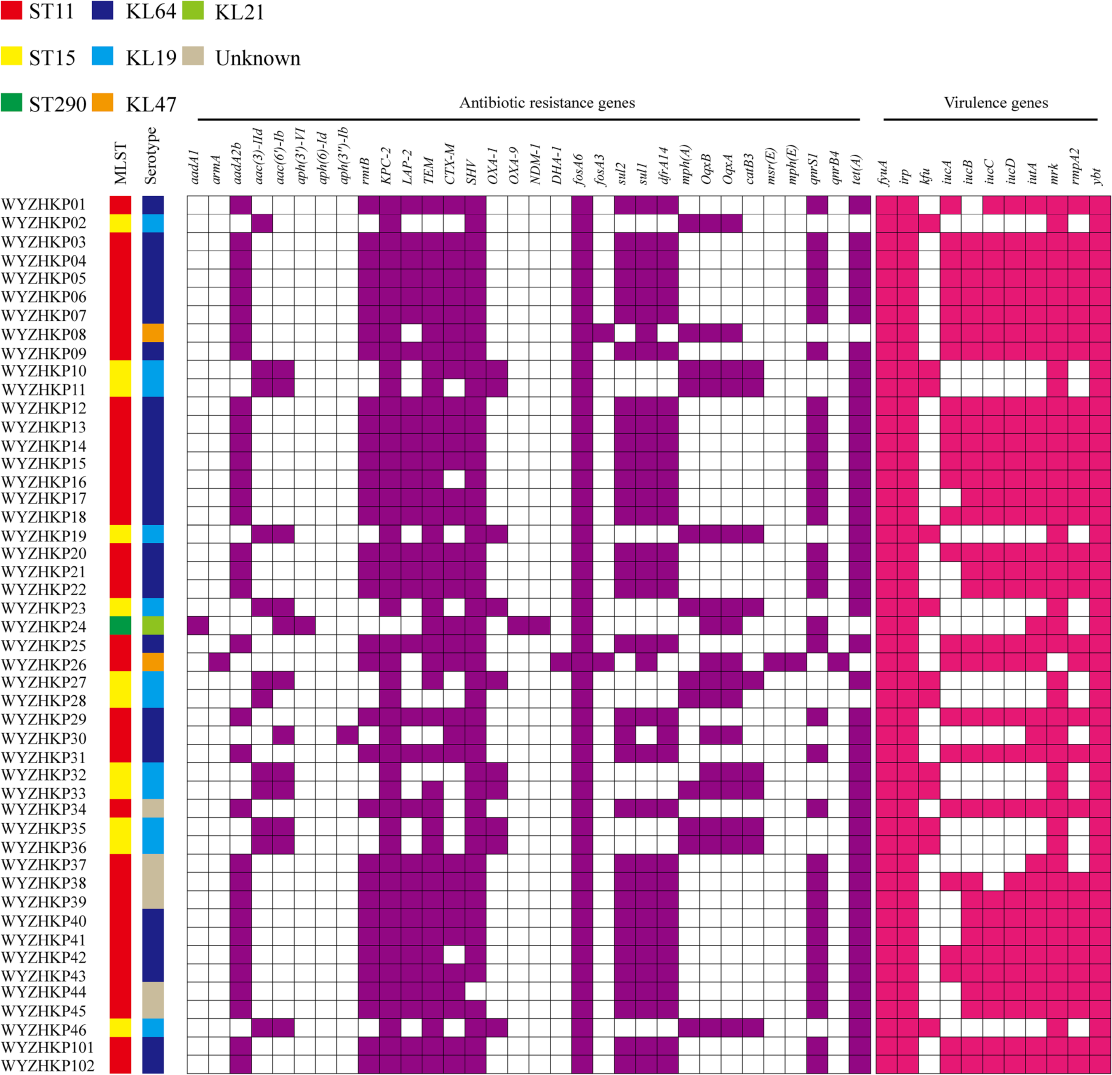
Virulence genes, antimicrobial resistance genes, capsular serotypes and MLSTs of 46 fecal colonization strains of CRE strains and 2 environmental CRE strains. The different MLSTs and capsular serotypes are color-coded and illustrated at the tips. The occurrence of antimicrobial resistance genes and virulence genes was also color coded.

A total of 46 CRKP-FC strains were subsequently subjected to whole-genome sequencing. Two strains of CRKP, WYZHKP101 and WYZHKP102, were isolated from the ICU for comparison with 46 strains of CRKP-FC, and whole-genome sequencing was performed. MLST analysis revealed that 46 CRKP-FC strains belonged to 3 different STs, with ST11 being the predominant ST (71.7%, 33/46), followed by ST15 (26.1%, 12/46) and ST290 (2.2%, 1/46) (Figure 2). Capsular serotypes revealed four different K loci; KL64 was the predominant capsule type (54.3%, 25/46), followed by KL19 (26.1%, 12/46), KL47 (4.3%, 2/46), and KL21 (2.2%, 1/46), and six strains could not be typed (Figure 2). These findings suggest that ST11-KL64 CRKP has emerged as the most prevalent CRKP-FC and may contribute to hospital outbreaks of infection.

The presence of carbapenemase genes is concerning, as they can cause resistance to many types of antibiotics, including carbapenems. All the ST11 and ST15 strains harbored *bla*_KPC-2_, and 10 ST15 strains carried two carbapenemase genes (*bla*_KPC-2_, *bla*_OXA-1_) at the same time (Figure 2). One ST290 strain carried two carbapenemase genes (*bla*_NDM-1_ and *bla*_OXA-9_) (Figure 2). Compared with ST11 strains, ST15 strains carried multiple carbapenemase genes, which further increased antibiotic resistance.

Almost all the ST11 strains carried the virulence genes *iuc*ABCD-*iut*A and *rmp*A2, whereas the ST15 strains lacked these virulence genes (Figure 2). ST11 strains carry more virulence genes than ST15 strains do, which is the opposite of resistance. This result suggests that strains with more resistance may have less virulence potential.

WYZHKP101 and WYZHKP102, which were isolated from the ICU environment, belong to the ST11-KL64 clone. The antibiotic resistance gene profile and virulence gene profile of these strains were similar to those of ST11-KL64 CRKP-FC (Figure 2). These findings suggest that the ST11-KL64 CRKP strains may cause nosocomial infection outbreaks.

### 3.6 Phylogenetic analysis of colonized CRKP

**Figure 3:**
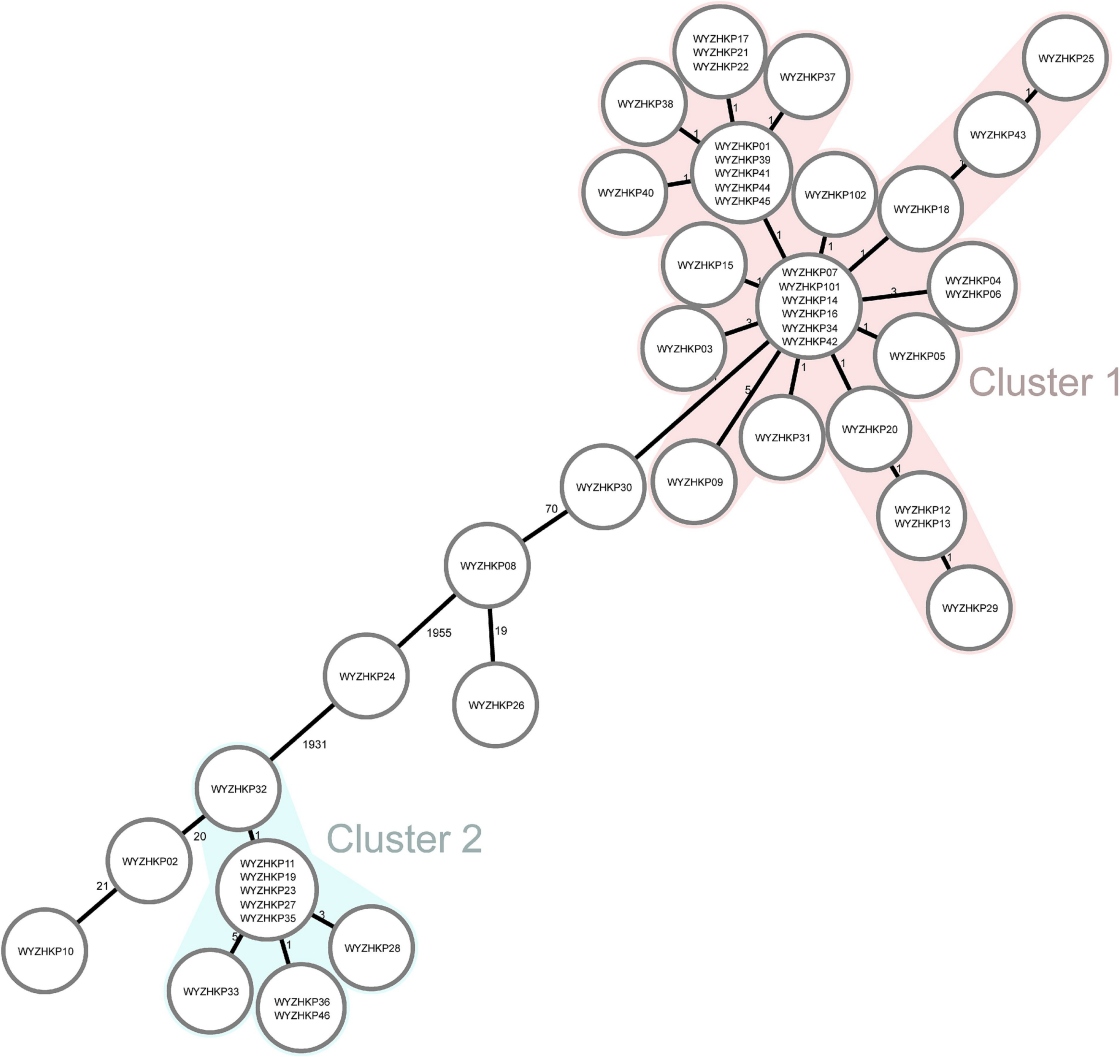
Minimum-spanning tree of cgMLST profiles among 46 fecal colonized CRE strains and 2 environmental CRE strains. The minimum-spanning tree was generated on the basis of cgMLST analysis with 2,358 conserved genome-wide genes. A cluster was defined at a distance of ≤15 alleles.

To gain a deeper understanding of the homology of these strains, we conducted whole-genome sequencing and core-genome phylogenetic analysis (Figure 3). The phylogenetic tree identified two major clades, each belonging to the same ST; specifically, cluster 1 corresponded to ST11, and cluster 2 corresponded to ST15 (Figures 2, 3).

The results revealed that the colonized ST11-KL64 CRKP strains dominated and formed a single cluster (Cluster 1) in ICU wards, indicating clonal expansion. These ST11 strains contained mainly KL64 capsular serotypes and a few other capsular serotypes (Figure 2). WYZHKP101 and WYZHKP102, which were isolated from the ICU environment, were ST11-KL64 clones belonging to Cluster 1. These findings suggest that ST11-KL64 CRKP has emerged as the most prevalent colonized carbapenem-resistant *K. pneumoniae* and may contribute to the outbreak of nosocomial infection. ST15 *K. pneumoniae* was identified as the second most prevalent colonized CRKP clone in ICU wards after ST11 *K. pneumoniae*. These ST15 strains contained only one capsule serotypes, KL19. These findings suggest that the ST15-KL19 CRKP strains formed a single cluster (Cluster 2), which may also have contributed to the outbreak of nosocomial infection.

To investigate the transmission of ST11-KL64 and ST15-KL19 *K. pneumoniae*, we conducted single-nucleotide polymorphisms (SNPs) based phylogenetic tree. The phylogenetic analysis included 48 *K. pneumoniae* genomes from this study and 185 *K. pneumoniae* genomes (ST11-KL64 and ST15-KL19) obtained from NCBI (Supplementary Table S1). A total of 185 *K. pneumoniae* (ST11-KL64 and ST15-KL19) from different countries and different isolation times were selected as reference strains.

**Figure 4:**
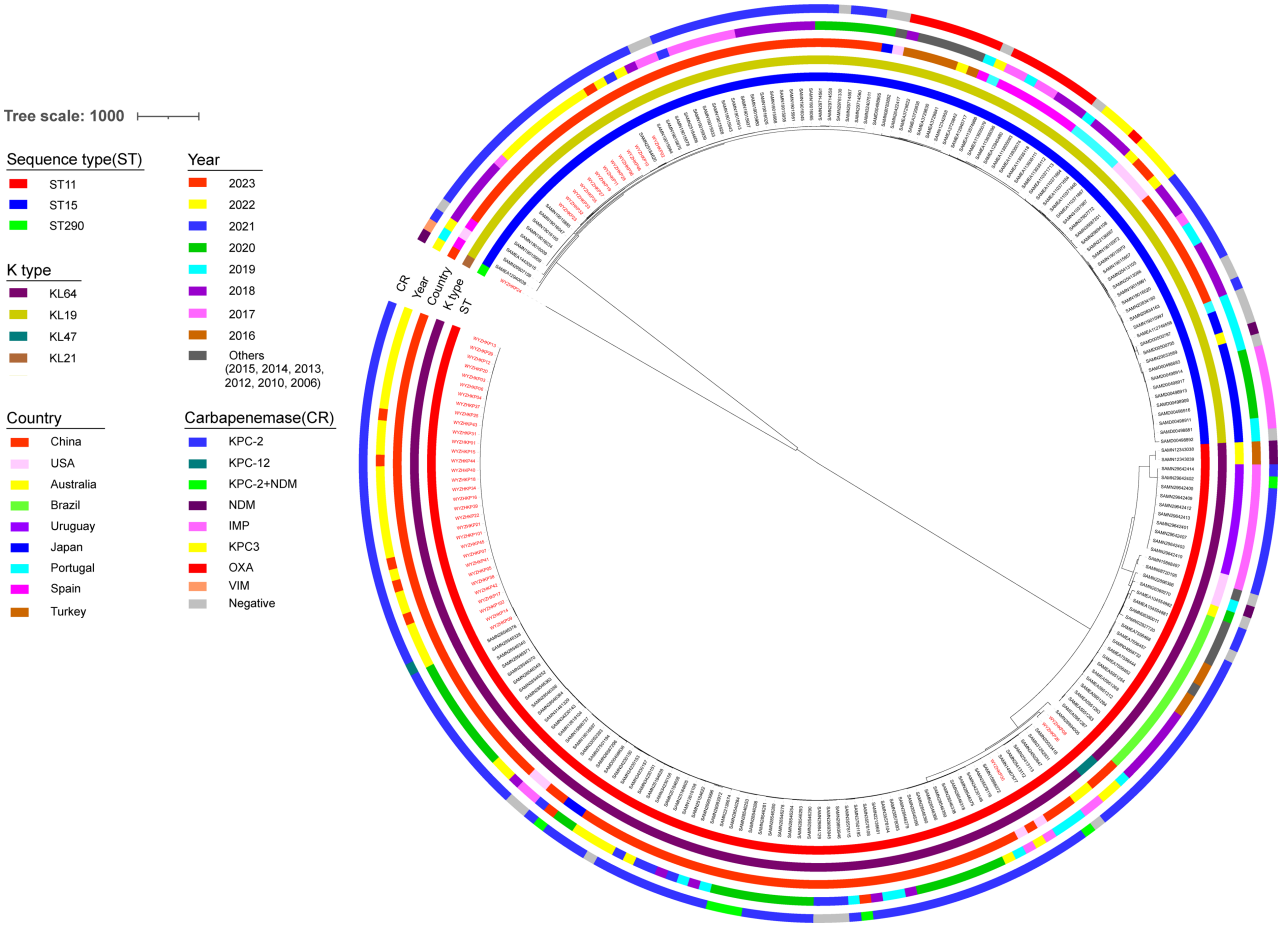
Phylogenetic analysis of 48 local CRKP strains and 185 reference strains. Maximum likelihood phylogenetic tree constructed by core genes of 233 *K. pneumoniae* isolates. The circles from the inner to the outer represent MLST, K type, country, year, and carbapenemase, respectively. The 48 local *K. pneumoniae* strains were marked in red.

As shown in Figure 4, the resulting phylogenetic tree had two main branches. ST11-KL64 and ST15-KL19 *K. pneumoniae* occupied two main branches, respectively. Clusters of SNPs based phylogenetic trees were similar to those of Minimum-spanning trees of cgMLST. Although in the same main branches, *K. pneumoniae* with the same MLST have obvious genetic distance if they come from different countries or have different isolation times. For the 48 strains in this study, *K. pneumoniae* with the same MLST had closer genetic distance if the strains were isolated in the same year.

## 4 Discussion

The CRE has recently become an extremely severe health challenge and public concern worldwide because of its high morbidity and mortality rates (2, 30). Fecal colonization by CRE is one of the risk factors for CRE infection in hospitalized patients (31). Screening for fecal colonization of CRE in ICU wards can contribute to better infection control measures to limit CRE dissemination. This prospective observational study aimed to investigate the molecular epidemiological characteristics and homology analysis of CRE fecal colonization in ICU wards of a university hospital in China.

This study revealed that the overall prevalence of CRE-FC in ICU wards was 12.6% (55/435). The predominant CRE-FC was CRKP, accounting for 83.6% (46/55) of the strains. In different studies around the world, the prevalence of CRE in fecal specimens varies from country to country. Some studies reported a lower prevalence of CRE-FC than the present study (32–35), whereas others reported a higher prevalence of CRE-FC (36, 37). The studies by Ogunbosi et al. (33) and Xu et al. (34) utilized pediatric fecal specimens, which may account for their reported lower prevalence of CRE fecal carriage compared with the present study. The fecal samples in the studies by Liu et al. (32) and Chu et al. (35) were not restricted to the ICU but also included those from other wards, which likely contributed to the lower prevalence of CRE fecal carriage than that found in our study. A higher prevalence of CRE fecal carriage reported by Mohan et al. in their study of hospitalized patients in India may be explained by differences in local infection control policies (37). The higher prevalence of CRE fecal carriage reported by Jaiswal et al.(36) in their study of patients with hematological malignancies is potentially due to the compromised immunity inherent to this patient group.

The irrational use of antibiotics and cross infection of patients in the hospital setting may lead to the high prevalence of CRE-FC. This study revealed that active screening of fecal colonization with CRE (CRE-FC) and IPC interventions could reduce the CRE infection rate in ICU wards. A 3-year prospective study in China suggested that active rapid molecular screening and other IPC interventions may significantly reduce the number of CRE nosocomial infections, even in wards without enough single-room isolation (21). A prospective study in South Korea suggested that active surveillance testing for carbapenem-resistant gram-negative bacteria may reduce their acquisition of clinical specimens in the ICU without additional costs (38). An Israeli study revealed that active screening and implementation of interventions led to a 34% reduction in CRE carriage over 3 years (39). What is the reason for the decrease in CRE infection rates through active screening and IPC interventions? The phylogenetic tree identified two major clades, each belonging to the same ST; specifically, cluster 1 corresponded to ST11, and clusters 2 to ST15. This study revealed that the colonized ST11 CRKP strains dominated and formed a single cluster (Cluster 1) in ICU wards, indicating clonal expansion. WYZHKP101 and WYZHKP102, which were isolated from the ICU environment, were ST11-KL64 clones belonging to Cluster 1. These findings suggest that the high rate of CRE infection in ICU wards is largely due to the clonal spread of CRE from patient to patient and from the environment to patient. Exploring the molecular epidemiological characteristics and homology analysis of fecal colonization of CRE in ICU wards can provide evidence for CRE transmission, which is highly valuable for improving infection control measures and reducing CRE infection rates. Our research was executed via a tripartite collaboration between the clinical department, the department of infection control, and the microbiology laboratory. The workflow involved active CRE screening, where ICU nurses collected patient stool specimens for analysis by the microbiology lab. Identified carriers were isolated with concomitant environmental decontamination and enforced hand hygiene. To assess intervention efficacy, the Infection Control team collected environmental specimens based on surveillance data, which were then analyzed by the microbiology lab to verify disinfection success, thereby aiming to block transmission routes and isolate infection sources. Given that CRE-FC in the ICU is primarily due to clonal spread, active fecal screening and enhanced IPC measures are crucial for reducing colonization and infection rates in ICU wards.

This study revealed that the predominant CRE-FC was CRKP, accounting for 83.6% (46/55) of the strains. Some studies have shown that CRKP accounts for 60–90% of clinical CRE infections in the United States, Europe, and China (3–5). Moreover, some studies have shown that colonization by CRE is dominated by CRKP (7, 8). These findings suggested that CRKP was dominant in both clinical CRE infection and intestinal CRE colonization. In this study, we further performed phenotypic and genomic analyses of 46 CRKP strains to prevent the spread of nosocomial infection.

With the widespread clinical use of carbapenems, the resistance of Enterobacteriaceae to carbapenems, particularly CRKP, has become increasingly serious in China (40, 41). In recent years, an alternating trend of subclones has been observed among the prevalent ST11 CRKP strains in China, with a significant increase in KL64 and a significant decrease in KL47 (6, 42, 43). ST11-KL64 CRKP strains carry a series of virulence genes associated with high pathogenicity, resulting in increased mortality in infected patients (42). In summary, many studies have investigated the molecular epidemiological characteristics of CRKP in clinical infection, but few studies have investigated the molecular epidemiological characteristics of fecal colonization of CRKP. This study revealed that the ST11-KL64 CRKP strain has emerged as the most prevalent CRKP-FC strain and may contribute to hospital outbreaks of infection. This finding is consistent with the clinical finding that ST11-KL64 CRKP is the most prevalent CRKP.

The limitations of this study are that it was a single-center study with a small number of strains. The generalizability of our findings to other hospital settings may be compromised by variations in local antibiotic stewardship policies and patient demographics. This study highlights the need for further studies on the relationship between fecal colonization by CRE and clinical infection with CRE. To address these limitations, we plan to conduct a multi-center study with a larger sample size to improve generalizability of our findings. This future work will concurrently collect both clinical CRE infection strains and fecal colonization strains from ICU patients. The primary objectives are to definitively investigate the relationship between CRE colonization and subsequent infection, and to identify the independent risk factors for this progression and elucidate the underlying evolutionary mechanisms.

## 5 Conclusion

The prevalence of fecal colonization by CRE (CRE-FC) in ICU wards was 12.6% (55/435), and CRKP was the main type of CRE-FC (83.6%, 46/55). Phylogenetic analysis revealed the clonal spread of CRKP among patients in ICU wards. ST11-KL64 CRKP has emerged as the most prevalent fecal colonizer of CRKP and may contribute to hospital outbreaks of infection. The active screening of CRE-FC and the implementation of IPC interventions can effectively reduce the CRE infection rate in ICU wards.

## Data Availability

The datasets presented in this study can be found in online repositories. The names of the repository/repositories and accession number(s) can be found at: https://www.ncbi.nlm.nih.gov/, PRJNA1100431.

## Footnotes

^1^ https://cge.food.dtu.dk/services/ResFinder/

^2^ https://bigsdb.Pasteur.fr/cgi-bin/bigsdb/bigsdb.pl?db=pubmlst_klebsiella_seqdef&page=sequenceQuery

^3^ https://github.com/nickjcroucher/gubbins

^4^ https://github.com/tseemann/snippy

^5^ https://itol.embl.de/
